# Effects of Dietary Additives on Nitrogen Balance, Odor Emissions, and Yolk Corticosterone in Laying Hens Fed Low-Protein Diets

**DOI:** 10.3390/ani15142021

**Published:** 2025-07-09

**Authors:** Ju-Yong Song, Yun-Ji Heo, Jina Park, Hyun-Kwan Lee, Yoo Bhin Kim, Byung-Yeon Kwon, Da-Hye Kim, Kyung-Woo Lee

**Affiliations:** Department of Animal Science and Technology, Konkuk University, 120 Neungdong-ro, Gwangjin-gu, Seoul 05029, Republic of Korea; 0jysong97@gmail.com (J.-Y.S.); bye0604@naver.com (Y.-J.H.); jinaa97@konkuk.ac.kr (J.P.); leehyun3177@naver.com (H.-K.L.); ybin51@naver.com (Y.B.K.); byung64@konkuk.ac.kr (B.-Y.K.); kim142536@naver.com (D.-H.K.)

**Keywords:** probiotics, exogenous enzymes, essential oils, saponins, corticosterone, stress

## Abstract

The objective of this study was to evaluate the effects of various feed additives on odor emissions, gut health, and stress responses in laying hens fed low-protein diets. Four commercially available functional feed additives (*Bacillus subtilis*, protease, saponin, and thyme-based essential oil) were selected for this study. A total of 288 Hy-Line brown laying hens aged 49 weeks were randomly fed on one of six experiment diets: a 16% standard crude protein diet, a 12% low-crude-protein (LCP) diet, and LCP diets supplemented with Bacillus-based probiotic, protease, saponin, or thyme-based essential oils prepared for 8 weeks. Each treatment had eight replicates with six birds per replicate. Lowering crude protein levels affected the laying performance, nitrogen balance, odor production (i.e., ammonia), and nutrient digestibility but did not alter eggshell quality or fecal short-chain fatty acids. Dietary additives added into the LCP diet did not affect the laying performance, egg qualities, and nitrogen balance but increased crude ash digestibility compared with the LCP-diet-fed laying hens. Branched-chain fatty acids tended to be higher in all laying hens fed low-CP diets, irrespective of feed additives. Notably, low vs. standard protein diets tended to increase yolk corticosterone levels, which is an indicator of stress responses in chickens. This low-CP-mediated increase in yolk corticosterone was partially decreased by 20.8–48.6% on average, depending on the additives used. Our study suggests that low-protein diets could effectively lower nitrogen excretion and odor emissions. However, adding dietary additives into low-protein diets has minimal effects on low-CP-diet-fed laying hens, which needs further studies to clarify the role of low-crude-protein diets and dietary additives in modulating hindgut fermentation via shaping the gut microbiota and stress responses of laying hens.

## Simple Summary

Low-protein diets have been practiced to lower nitrogen excretion and odor emissions while maintaining laying performance and egg quality in the poultry industry. Recently, we have addressed that reductions in nutrition (i.e., crude protein levels) impair gut health and induce physiological stress responses, including the release of corticosterone, although laying hens still perform well. In this study, we explored whether feed additives affecting gut health or improving feed-origin nutrients might modulate nutrition-related stress responses in laying hens fed low-protein diets. Here, we provided evidence that dietary additives can be used to help laying hens cope with nutritional stress.

## 1. Introduction

Excessive protein intake can result in a substantial release of nitrogen, generating odors through the excretion of uric acid in poultry, which is considered the most influential environmental problem in the poultry industry [1]. Undigested excreta emit various gases, including amines, carbon dioxide, hydrogen sulfide, and ammonia, some of which affect global warming [2]. The best strategy to mitigate nitrogen excretion is to decrease the protein content of chicken diets [3]. However, feeding a chicken a low-protein diet could lead to a decline in the performance of broiler chickens [4] and laying hens [5]. Reducing protein levels for environmental reasons is not a relevant nutritional solution because of the positive correlation between protein intake and improved growth and production performance [6]. Therefore, it is crucial to provide an appropriate amount of protein to maintain production performance and minimize odor issues in livestock [7].

Thus, it is of the utmost importance to maintain the efficiency of digestion and absorption of nutrients as much as possible. However, it has been reported that low-protein diets can impair gut functions, such as villus morphology [8]. Therefore, adding dietary additives to low-protein diets is expected to improve the low-protein-diet-induced reduction, if any, in performance and intestinal health. Previous studies have shown that exogenous proteases improve gut health by increasing villus height or tight junction proteins in broiler chickens [9]. Dietary *Bacillus subtilis* has been utilized as a probiotic candidate to improve gut function in laying hens [10]. Dietary saponins are known to alter the ileal gut microflora and improve systemic immune function in laying hens [11]. Essential oils are aromatic oily liquids obtained from plant materials that improve gut function, including the integrity and absorptive capacity of the intestine in laying hens [12]. It has been reported that the laying performance and egg quality in laying hens can be improved by supplementation with *Bacillus subtilis* [13], exogenous proteases [14], saponins [11], and oregano essential oil [15]. In addition, earlier studies have reported that dietary additives counteracted the negative effects of low-protein diets (1% or 2% reduction in crude protein compared with the standard control diet) on the laying performance or gut health in laying hens [16,17]. To the best of the authors’ knowledge, it has not been studied whether dietary additives can improve the health and performance of laying hens fed very-low-protein diets (e.g., over 4%). In our earlier study [1], pullets fed on graded CP diets from 12 to 18% CP exhibited a linear decrease in the ileal villus height. In addition, laying hens (aged 19 to 32 weeks) fed on graded CP diets from 13 to 19% linearly lowered the egg weight but linearly increased the Haugh unit [1]. Based on the earlier study [1], we decided to use 12% low-CP diets fortified with essential amino acids that might affect gut health, as shown in pullets [1]. Thus, the objective of this study was to determine the effect of dietary additives on the laying performance, egg quality, gut health, nitrogen excretion, and odor emission of laying hens fed very-low-crude-protein diets. In addition, we evaluated whether low-protein diets could be considered one of the nutritional stress factors and, if so, whether dietary additives could reverse low-protein-diet-induced nutritional stress in laying hens. It is hypothesized that dietary additives would lower odor emissions, mitigate stress responses, and improve the gut health of low-CP-diet-fed laying hens.

## 2. Materials and Methods

### 2.1. Animal Care

The experimental procedures were approved by the Institutional Animal Care and Use Committee of Konkuk University (KU22033).

### 2.2. Animals, Diets, and Experimental Design

A total of 288 Hy-Line Brown laying hens aged 49 weeks were raised in a windowless fan-ventilated chicken facility for 8 weeks. Two hens were housed per cage (45 cm × 45 cm), and three adjacent cages were considered replicates (*n* = 6 birds/replicate, 8 replicates/treatment). Laying hens were randomly subjected to one of six experimental diets (Table 1): 16% crude protein (CP) standard diet (SCP), 12% CP low diet (LCP), and the LCP diet supplemented with *Bacillus*-based probiotics (300,000 CFU/kg; Enviva Pro, Dupont-Danisco, Palo Alto, CA, USA), protease (10,000 U/kg; Pearlzyme, PEARLZYME CO., Inchon-si, Republic of Korea), saponin (200 mg/kg; *Quillaja* saponin-enriched biomass powder, Delacon Inc., Steyregg, Austria), or thyme- and star anise-based essential oil preparation (150 mg/kg; BIOSTRONG 510, Delacon Inc., Steyregg, Austria). All additives were commercially available, and their inclusion levels were determined according to the manufacturer’s recommendations.

It is reported that those additives used in this study have been used to improve the performance of chickens [9,10,11,12,13,14,15]. Corn- and soybean-meal-based SCP and LCP control diets were formulated (Table 2) to contain equal apparent metabolizable energy and limiting amino acids (lysine, methionine, and threonine). The analyzed chemical composition of the control diets, including essential amino acids, is presented in Table 2.

Laying hens were exposed to an 18 L:6 D lighting schedule. Food and water were provided ad libitum. The temperature was set as 21 °C with a relative humidity of 60%.

### 2.3. Sample Collection and Egg Quality Measurements

Body weight and feed intake were measured at the beginning and end of the experiment. The eggs were collected and weighed daily to assess their productivity and egg weight. Egg mass was calculated as egg production (%) multiplied by egg weight (g/egg), and the feed conversion ratio was calculated as the feed intake divided by egg mass. Eggs (three per replicate) were sampled for egg quality measurements on three subsequent days at weeks 4 and 8 of the experiment. A color reflectometer (TSS QCR; Technical Services and Supplies, York, UK) was used to measure the eggshell color, and a digital egg tester (DET-6000; Nabel, Kyoto, Japan) was used to measure the egg quality, including the yolk color, Haugh unit, and eggshell thickness.

### 2.4. Determination of Nutrient Digestibility and Odor Emission

The total fecal collection method was used to measure nutrient digestibility. At the end of the experiment, four hens per replicate were moved into metabolic cages and adapted for three days. After the adaptation, total excreta were quantitatively collected for 3 days and weighed and dried at 65 °C for 3 days. Diets and dried excreta were ground and analyzed [18,19] for dry matter (method 930.15), CP (method 990.03), ether extract (method 920.39), ash (method 942.05), and neutral detergent fiber (NDF).

Approximately 150 g of freshly voided excreta were collected immediately after total fecal collection to measure odor emissions. The collected feces were placed in a 6 L plastic box, and nitrogen gas was injected at 1 L/min for 10 min to achieve concentration equilibrium of the odor. The amounts of ammonia, hydrogen sulfide, trimethylamine, and carbon dioxide were measured and recorded using a gas-sampling pump kit (Gastec Corp, Tokyo, Japan).

### 2.5. Volatile Fatty Acids Analysis

At the end of the experiment, 1 g of freshly voided excreta was collected to measure short-chain fatty acids. The collected digesta were homogenized with pivalic acid, 25% H_3_PO_4_, HgCl_2_, and distilled water and centrifuged at 3000 rpm for 20 min. The separated supernatant was used to measure short-chain fatty acids using gas chromatography (6890 series GC system; HP, Palo Alto, CA, USA), as described previously [20].

### 2.6. Corticosterone in Egg Yolk

Three eggs per replicate were collected for corticosterone determination at weeks 4 and 8. The eggs were cracked open, and the yolk was separated from the albumen and pooled. The pooled yolk per replicate was homogenized, and the corticosterone concentration was measured using a CORT ELISA kit (Enzo Life Science Inc., ADI-901-097, Farmingdale, NY, USA), as described previously [21,22].

### 2.7. Statistical Analysis

Data were analyzed with ANOVA using the GLM procedure in SAS (version 9.4; SAS Institute Inc., Cary, NC, USA). Dietary treatment was a fixed factor in all statistical models. Tukey’s test was used to determine differences among treatments. The significance level was set at *p* < 0.05.

## 3. Results

Feed intake and egg production did not differ (*p* < 0.05) between dietary treatments (Table 3). Laying hens fed on the LCP diet tended to lay lighter eggs by 4.0% compared with those on the SCP diet (*p* = 0.055). Consequently, the LCP group had a lower egg mass compared with the SCP group (*p* < 0.001). Dietary supplementation with additives into the LCP diet did not significantly improve the egg weight and egg mass compared with the LCP diet (*p* > 0.05). Dietary saponin tended to slightly increase the egg weight by, on average, 3.4% compared with the LCP group. The feed conversion ratio was lowest in the SCP-diet-fed control group, and the diet supplementation with additives did not have a positive effect on the feed conversion ratio. The final body weight was not altered (*p* = 0.143) between dietary treatments (Table 3).

Neither CP levels nor dietary additives affected the eggshell strength and Haugh units at 4 and 8 weeks (Table 4). There were no significant differences in eggshell thickness or color at 4 weeks. Yolk colors of the SCP group remained darker than those in the other groups at 4 and 8 weeks (*p* < 0.001). Eggshell thickness at 8 weeks (but not at 4 weeks) was the lowest in saponin-fed laying hens (*p* < 0.05). Interestingly, the eggshell color was not different (*p* > 0.05) between the SCP and LCP groups but was darker (*p* < 0.05) in saponin-fed vs. LCP-fed laying hens at 8 weeks.

Although the LCP group lowered the digestibility of dry matter, CP, crude fat, and crude ash by an average of 8.4%, 20.0%, 7.5%, and 49.4%, compared with the SCP group, a statistically significant difference was noted only for crude ash digestibility (Table 5). Dietary additives added to the LCP diet did not significantly affect (*p* > 0.05) nutrient digestibility, except for that of crude ash (Table 5).

As expected, the nitrogen intake was low (*p* < 0.05) in the LCP vs. SCP diets (Table 6). Adding dietary additives to the LCP diet tended to increase or decrease the nitrogen intake depending on the additives used (*p* > 0.05). Nitrogen excretion exhibited a similar pattern to the nitrogen intake, indicating a lower nitrogen intake and excretion. Consequently, nitrogen retention was the highest in the SCP treatment, and no differences were noted between the LCP and dietary additives. The excreta output (g/d/hen) and moisture contents in excreta were not different between treatment groups (Table 6).

No differences were noted in the concentrations of acetate, propionate, or butyrate in the excreta samples of the LCP and SCP groups (Table 7). The concentrations of isovalerate, valerate, and branched-chain fatty acids were highest in the protease-supplemented-diet-fed laying hens. The concentrations of total short-chain fatty acids were not different between dietary treatments (*p* > 0.05).

None of the dietary treatments altered (*p* > 0.05) the corticosterone content in egg yolk samples at 4 weeks (Table 8). However, the LCP vs. SCP diets tended to increase (*p* > 0.05) the corticosterone concentration by 51.2% at 8 weeks. Of interest, the yolk corticosterone levels of laying hens fed on dietary additives tended to be lower by, on average, 20.8% to 48.6% compared to the LCP group (*p* > 0.05).

None of the dietary treatments affected the hydrogen sulfide and carbon dioxide concentrations (Table 9). However, the concentrations of ammonia and trimethyl amine in fresh excreta of the LCP group were significantly lower compared to those of the SCP group (*p* < 0.05). None of the dietary additives affected trimethylamine production (*p* > 0.05); however, the concentration of ammonia was elevated (*p* < 0.05) in laying hens fed diets containing saponins and essential oils compared with the LCP group.

## 4. Discussion

It appeared that lowering CP levels did not affect the laying performance except for the egg mass compared with the standard-CP diet. Consequently, adding dietary additives to the LCP diet did not affect the laying performance. The lack of a low-CP diet could be partly related to the equal-limiting amino acids in SCP and LCP diets. Indeed, earlier studies [23,24] showed that supplementing the LCP diet with synthetic amino acids exhibited an equal performance to that with the SCP diet. Nonetheless, it should be kept in mind that laying hens fed diets containing LCP vs. SCP exhibited a moderate reduction in egg weight and egg mass. Furthermore, this LCP-mediated reduction in egg weight and egg mass was not recovered by dietary feed additives (e.g., *Bacillus subtilis*, protease, saponin, and essential oils). It was noted that dietary saponin tended to increase the egg weight by 3.4% compared with the LCP group. This numerical increase in egg weight may be due to a numerical increase in feed intake (see Table 2) or in the nutrients’ digestibility (see Table 5) in the saponin group vs. the LCP group. Further studies are required to test whether dietary additives added to the LCP diet formulated with less-digestible ingredients without fortification with limiting amino acids can improve the performance of laying hens.

Egg quality is closely associated with genetic factors, diet, health, and the environment [25]. Our study showed that LCP vs. SCP diets or dietary additives added to the LCP diet did not affect egg quality, except for the yolk color. It has been reported that the intake of pigmented substances such as carotenoids largely influences yolk color. The pale yolk color observed in this study was related to the low levels of corn gluten meal in the LCP vs. SCP diets, as reported elsewhere [26]. Although eggshell thickness was lowest in laying hens fed a diet containing saponins, this finding needs careful explanation, as eggshell strength was not altered among dietary treatments. Surprisingly, eggshell color, measured using a reflectometer, was darker in eggs laid by saponin-fed laying hens than by those in the LCP groups at 8 weeks. Eggshell color is influenced by various factors, including aging, nutrients (e.g., antioxidants, trace minerals, probiotics, and essential oils), stress, and disease status, all of which affect pigment synthesis [27]. At this stage, clear explanations on whether dietary saponins would affect the pigment synthesis pathway (i.e., protoporphyrin IX) in the eggshell glands of laying hens are not readily available. In contrast, Mao et al. [28] reported that saponin-rich *Yucca schidigera* extract did not affect the eggshell color in laying hens. Also, the eggshell color noted in this study was within the normal range, from 24% to 30% [29].

Consistent with our findings, Heo et al. [1] reported that crude ash digestibility decreased with increasing dietary CP levels in laying hens. Thus, the partial reduction in the digestibility of dry matter noted in LCP vs. SCP was attributed to a concomitant decrease in ash digestibility. Among the additives tested, dietary essential oils had a marked impact on crude ash digestibility compared to the LCP. This pattern was also observed in the digestibility of dry matter, CP, and crude fat, although the differences were not statistically significant. Previous studies have reported that dietary essential oils increase digestive secretion and enzyme activity, leading to nutrient absorption at the gut level [30]. It should also be noted that eggshell quality, including thickness and strength, was not altered, although dietary additives, especially in the essential oil group, increased the LCP-mediated decrease in crude ash digestibility. Further analysis of digestive enzyme activity and/or the digestibility of minerals (i.e., calcium, phosphorus, or magnesium) may explain the improved ash digestibility in laying hens fed on dietary essential oils.

It is well understood that lowering CP levels is an effective strategy to minimize nutrient load from poultry manure. However, it is not known whether lowering CP levels could influence fecal mass production, which urged us to quantify fecal output. We found that fresh excreta tended to be higher in laying hens fed the LCP diet as well as in those fed dietary additives than in the SCP diet group. In contrast, the dry matter content in excreta tended to be lower in the LCP- and additive-fed groups than in the SCP group, indicating a higher moisture content in these groups. Whether higher moisture content results from higher water intake needs to be addressed. Our study also confirmed the notion [1] that lowering CP levels in the diets of laying hens is the most effective strategy for lowering nitrogen excretion. In addition, dietary additives added to the LCP diet did not affect the nitrogen balance. This could be related to the current experimental design, in which the LCP had extremely low-CP levels and highly digestible diet ingredients (e.g., soybean meal or corn gluten meal). Thus, if less-digestible protein ingredients, such as feather meal, cottonseed meal, meat, and bone meal [31,32] are used in low-protein diets, direct or indirect actions by the additives on the less digestible substrates at the gut level might lead to improved nutrient digestibility.

The purpose of determining the volatile fatty acids in laying hen feces was to identify and measure intestinal microbial fermentation products, as low-protein diets could impair gut functions, including gut morphology and gut microbiota [1,33]. Volatile fatty acids such as acetate, propionate, or butyrate are one of the main metabolites generated from the fermentation of carbohydrates by intestinal commensal bacteria; therefore, they can be used as indicators of the composition of beneficial intestinal microorganisms [34]. However, no significant differences between dietary treatments were noted in the concentrations of fecal short-chain fatty acids. In contrast, the concentrations of branched-chain fatty acids tended to be higher in laying hens fed on the LCP diet and the LCP diets enriched with additives (especially the protease group) compared with the SCP-diet-fed group. As branched-chain fatty acids are essentially produced from branched-chain amino acids by cecal microflora [35], our finding might suggest that undigested protein sources (e.g., branched-chain amino acids) originating from diets or the host may be increased in hens fed on low-CP diets irrespective of the additives used. Indeed, we found that NDF digestibility was not altered, but CP digestibility tended to be low in all low-CP-diet-fed laying hens compared with the SCP group. It is thus likely that a low-CP diet per se may impair gut functions, including gut integrity, microbiota composition, or CP digestibility, leading to alterations in the end products of protein fermentation. In addition, it is known that exogenous proteases add digestive capacity to endogenous proteases, releasing incompletely digested feed-origin proteins [36]. Thus, it is likely that exogenous enzyme-released protein substrates could escape immediate absorption in the small intestine and become distally available for bacterial fermentation. In any event, our study warrants further clarification of individual amino acid digestibility and the composition of distal gut microflora in laying hens fed on standard or low-CP diets.

The stress hormone corticosterone decreases feed intake, weight gain, and immune function in laying hens [37]. Therefore, corticosterone levels in laying hens can significantly affect their growth and immunity. In the current study, LCP vs. SCP diets exhibited a 1.51-fold increase in yolk corticosterone levels at 8 weeks. Interestingly, dietary additives tended to decrease LCP-mediated increases in yolk corticosterone levels. Previous studies have shown that a nutritional imbalance can cause stress in animals [38]. Therefore, it is assumed that adding dietary additives, as observed in the current study, could mitigate the nutritional stress induced by nutrient-deficient diets. Indeed, Park et al. [39] reported that low vs. standard phosphorus diets increased yolk corticosterone in laying hens, but dietary phytase supplementation in the low-phosphorus diet reversed the low-phosphorus diet-mediated increase in yolk corticosterone. However, we should be cautious when interpreting the results, as the difference between the groups is statistically insignificant (*p* = 0.052), which warrants further studies.

Odors produced in livestock excreta are major environmental pollutants. Odors are mainly created through the fermentation of undigested carbohydrates or proteins by microorganisms [1,39,40]. In line with nitrogen pollution in the poultry industry, nutritional strategies to minimize odor production include lowering protein levels in diets and optimizing gut health using functional feed additives. Indeed, we found that LCP lowered odor production, as previously reported [41], confirming the nutritional approaches for mitigating nitrogen excretion in laying hens. However, a lack of effect of low-protein diets on ammonia emissions has also been reported in laying hens [24,42]. It is not clearly understood how the ammonia concentration that was elevated by dietary additives (especially saponins and essential oils) increased the LCP-mediated decrease in ammonia production.

## 5. Conclusions

The LCP vs. SCP diets lowered egg mass, but dietary additives added into the LCP diet failed to improve the performance of laying hens. The LCP diet decreased the digestibility of nutrients such as crude ash, but dietary additives (e.g., essential oil) increased an LCP-mediated decrease in crude ash digestibility. The LCP vs. SCP diets lowered the nitrogen intake and excretion. However, dietary additives had a minimal effect on the nitrogen balance. The LCP vs. SCP diets tended to increase yolk corticosterone, but dietary additives tended to alleviate this increase. The LCP diets lowered the ammonia concentration compared to the SCP diets. However, supplementation with saponins and essential oils increased the ammonia concentration compared to the LCP-diet-fed groups. Our study showed that dietary additives did not significantly affect the LCP-mediated effects in laying hens. However, further studies are warranted to clarify the gut microbiota or stress responses in laying hens fed low-CP diets and LCP diets enriched with feed additives.

## Figures and Tables

**Table 1:** Summary of experimental diets with crude protein (CP) and additives.

Treatments	CP (%) in Diets	Additives Used
Standard CP (SCP)	16	None
Low CP (LCP)	12	None
Probiotics	12	*Bacillus*-based probiotics
Exogenous enzyme	12	Serine protease
Saponin	12	*Quillaja* saponin
Essential oil	12	Thyme- and star-based essential oil

**Table 2:** Ingredients and composition of standard and low-crude-protein (CP) diets.

Items	Low-CP Diet (12% CP)	Standard-CP Diet (16% CP)
Ingredients composition, %		
Maize	70.16	61.04
Soybean meal, 47% CP	9.87	17.22
Tallow	2.00	2.67
Corn gluten meal	1.00	4.60
Maize distillers grain and solubles	2.30	0.75
Sodium chloride	0.22	0.24
Sodium bicarbonate	0.20	0.19
Limestone	10.70	10.65
Monocalcium phosphate	1.85	1.82
L-lysine HCl, 78.8%	0.62	0.23
DL-methionine, 99%	0.24	0.15
L-threonine, 99%	0.25	0.08
L-valine, 99%	0.33	0.11
Choline chloride	0.05	0.05
Vitamin premix ^1^	0.10	0.10
Mineral premix ^2^	0.10	0.10
Calculated chemical composition		
Dry matter, %	88.46	88.74
AMEn ^3^, kcal/kg	2750	2750
Crude protein, %	12.38	16.62
Lysine, %	0.80	0.80
Threonine, %	0.66	0.66
Met + Cys, %	0.73	0.73
Calcium, %	4.20	4.20
Available phosphorus, %	0.40	0.40
Crude ash, %	14.0	14.3
Ether extract, %	4.55	5.01

^1^ Vitamin premix provided following nutrients per kg of diet: vitamin A, 20,000 IU; vitamin D3, 4600 IU; vitamin E, 40 mg; vitamin K3, 4 mg; vitamin B1, 4 mg; vitamin B2, 8 mg; vitamin B6, 6 mg; vitamin B12, 0.04 mg; biotin, 0.24 mg; D-pantothenic acid, 18.37 mg; niacin, nicotinic acid, 60 mg, folic acid, folic acid, 1.4 mg, butylated hydroxytoluene, 0.25 mg. ^2^ Mineral premix provided the following nutrients per kilogram of diet: Fe, 70 mg; Mn, 80 mg; Zn, 60 mg; Cu, 8 mg; Co, 0.13 mg; I, 1 mg; Se, 0.20 mg. ^3^ Nitrogen-corrected apparent metabolizable energy.

**Table 3:** Effects of dietary additives on laying performance in laying hens fed on low-protein diets ^1^.

Items	Treatments						SEM	*p*-Value
	SCP	LCP	*B. subtilis*	Protease	Saponin	Essential Oil		
Feed intake, g/hen	119.2	117.5	118.9	119.4	119.6	119.6	0.52	0.053
Egg production, %	91.3	88.1	87.3	87.5	86.4	87.0	1.43	0.055
Egg weight, g	65.1 ^a^	62.5 ^abc^	60.3 ^c^	61.9 ^b^	64.6 ^ab^	61.9 ^bc^	0.73	<0.001
Egg mass, g/day	59.4 ^a^	55.1 ^b^	52.7 ^b^	54.2 ^b^	55.8 ^ab^	53.9 ^b^	0.85	<0.001
FCR, kg: kg	2.01 ^b^	2.13 ^ab^	2.26 ^a^	2.21 ^a^	2.14 ^ab^	2.22 ^a^	0.04	<0.001
Initial BW, kg/hen	1.90	2.00	1.90	2.01	2.00	1.91	0.04	0.439
Final BW, kg/hen	2.08	1.90	1.94	1.94	1.99	1.98	0.05	0.143
Soft and broken egg, %	0.0018	0.0009	0.0022	0.00	0.0009	0.00	0.0004	0.402

^1^ SCP, standard crude protein (CP); LCP, low-CP diet; SEM, standard errors of the mean; FCR, feed conversion ratio; BW, body weight. ^a,b,c^ Means with different superscripts within the same row differ significantly (*p* < 0.05).

**Table 4:** Effects of dietary additives on egg quality in laying hens fed on low-protein diets ^1^.

Items	Treatments						SEM	*p*-Value
	SCP	LCP	*B. subtilis*	Protease	Saponin	Essential Oil		
Eggshell strength, kgf								
4 weeks	4.99	4.83	5.02	4.99	4.85	5.20	0.15	0.600
8 weeks	4.75	4.93	5.4	4.84	5.13	5.24	0.18	0.093
Eggshell thickness, mm								
4 weeks	0.39	0.41	0.41	0.41	0.41	0.40	0.01	0.299
8 weeks	0.40 ^ab^	0.43 ^a^	0.42 ^a^	0.41 ^ab^	0.39 ^b^	0.41 ^ab^	0.01	0.004
Eggshell color								
4 weeks	26.43	29.40	28.73	26.65	26.57	28.59	0.92	0.086
8 weeks	26.29 ^a^	26.51 ^a^	25.26 ^ab^	23.66 ^ab^	21.65 ^b^	22.88 ^ab^	0.93	0.002
Yolk color, RCF								
4 weeks	8.64 ^a^	7.41 ^b^	7.80 ^b^	7.40 ^b^	7.78 ^b^	7.62 ^b^	0.10	<0.001
8 weeks	8.61 ^a^	7.25 ^b^	7.29 ^b^	7.30 ^b^	7.23 ^b^	7.25 ^b^	0.09	<0.001
Haugh unit								
4 weeks	90.30	93.20	91.8	93.70	92.40	92.80	0.81	0.074
8 weeks	92.33	93.30	93.13	90.78	92.47	90.81	1.14	0.463

^1^ SCP, standard crude protein (CP); LCP, low-CP diet; SEM, standard errors of the mean; RCF, Roche color fan. ^a,b^ Means with different superscripts within the same row differ significantly (*p* < 0.05).

**Table 5:** Effects of dietary additives on apparent total tract digestibility of nutrients in laying hens fed on low-protein diets ^1^.

Items	Treatments						SEM	*p*-Value
	SCP	LCP	*B. subtilis*	Protease	Saponin	Essential Oil		
Dry matter	73.6 ^a^	67.4 ^ab^	65.3 ^b^	72.0 ^ab^	67.3 ^ab^	71.3 ^ab^	1.8	0.026
Crude protein	46.6	37.3	36.0	42.7	34.6	44.1	3.7	0.166
Crude fat	86.6	80.1	81.9	82.8	80.6	83.0	1.5	0.079
Crude ash	43.3 ^a^	21.9 ^c^	23.4 ^bc^	33.7 ^abc^	31.7 ^abc^	36.1 ^ab^	3.1	<0.001
NDF	26.3	28.9	28.0	24.8	28.2	29.2	1.8	0.509

^1^ SCP, standard crude protein (CP); LCP, low-CP diet; SEM, standard errors of the mean; NDF, neutral detergent fiber. ^a,b,c^ Means with different superscripts within the same row differ significantly (*p* < 0.05).

**Table 6:** Effects of dietary additives on nitrogen (N) balance in laying hens fed on low-protein diets ^1^.

Items	Treatments						SEM	*p*-Value
	SCP	LCP	*B. subtilis*	Protease	Saponin	Essential Oil		
Fresh excreta (g/d/hen)	91.4	106.1	108.8	97.0	102.0	95.1	4.27	0.060
Moisture content in excreta (%)	68.3	78.6	78.9	72.7	74.8	71.4	3.11	0.159
N intake (g/d/hen)	2.63 ^a^	1.89 ^bc^	2.06 ^b^	1.90 ^bc^	1.86 ^bc^	1.82 ^c^	0.05	<0.001
N excretion (g/d/hen)	1.41 ^a^	1.18 ^ab^	1.32 ^ab^	1.09 ^ab^	1.21 ^ab^	1.01 ^b^	0.07	0.010
N retention (g/d/hen)	1.22 ^a^	0.71 ^b^	0.74 ^b^	0.81 ^b^	0.65 ^b^	0.81 ^b^	0.08	<0.001

^1^ SCP, standard crude protein (CP); LCP, low-CP diet; SEM, standard errors of the mean. ^a,b,c^ Means with different superscripts within the same row differ significantly (*p* < 0.05).

**Table 7:** Effects of dietary additives on fecal volatile fatty acid concentration (mmol/kg of feces) in laying hens fed on low-protein diets ^1^.

Items	Treatments						SEM	*p*-Value
	SCP	LCP	*B. subtilis*	Protease	Saponin	Essential Oil		
Acetate	16.03	14.46	24.62	12.31	16.03	19.99	3.623	0.234
Propionate	2.01	2.48	3.68	3.68	3.58	4.08	0.507	0.054
Butyrate	2.53	3.15	4.35	3.93	4.74	5.55	0.775	0.118
Isovalerate	0.79 ^b^	0.98 ^b^	1.22 ^ab^	2.22 ^a^	1.12 ^ab^	1.34 ^ab^	0.257	0.012
Valerate	1.10 ^b^	1.53 ^ab^	1.86 ^ab^	2.28 ^a^	1.57 ^ab^	1.63 ^ab^	0.202	0.012
BCFA	8.92 ^b^	12.19 ^ab^	10.01 ^b^	18.52 ^a^	10.34 ^ab^	10.19 ^b^	1.890	0.018
SCFA	22.5	22.6	35.7	24.4	27.0	32.6	4.700	0.256

^1^ SCP, standard crude protein (CP); LCP, low-CP diet; SEM, standard errors of the mean; SCFA, short-chain fatty acids (acetate + propionate + butyrate + isovalerate + valerate); BCFA, branched-chain fatty acids (valerate + isovalerate). ^a,b^ Means with different superscripts within the same row differ significantly (*p* < 0.05).

**Table 8:** Effect of dietary additives on yolk corticosterone (pg/g) in laying hens fed on low-protein diets ^1^.

Yolk Corticosterone	Treatments						SEM	*p*-Value
	SCP	LCP	*B. subtilis*	Protease	Saponin	Essential Oil		
4 weeks	309	301	234	213	256	193	93.46	0.941
8 weeks	121	183	125	111	145	94	19.38	0.052

^1^ SCP, standard crude protein (CP); LCP, low-CP diet; SEM, standard errors of the mean.

**Table 9:** Effects of dietary additives on odor emission (ppm) in laying hens fed on low-protein diets ^1^.

Items	Treatments						SEM	*p*-Value
	SCP	LCP	*B. subtilis*	Protease	Saponin	Essential Oil		
Ammonia	80 ^a^	40 ^c^	40 ^c^	40 ^c^	53 ^b^	51 ^b^	2.49	<0.001
Hydrogen sulfide	0.32	0.46	0.70	0.46	0.30	0.50	0.14	0.444
Trimethyl amine	90 ^a^	36 ^b^	31 ^b^	23 ^b^	27 ^b^	24 ^b^	3.12	<0.001
Carbon dioxide	340	380	420	380	350	420	64.8	0.924

^1^ SCP, standard crude protein (CP); LCP, low-CP diet; SEM, standard errors of the mean. ^a,b,c^ Means with different superscripts within the same row differ significantly (*p* < 0.05).

## Data Availability

The original contributions presented in this study are included in the article. Further inquiries can be directed to the corresponding author.
